# Working Memory Impairment Induced by Chemotherapy—A Narrative Review

**DOI:** 10.3390/brainsci16080805

**Published:** 2026-07-30

**Authors:** Nikola M. Stojanović, Mihajlo Marjanović, Milan N. Petković, Ivana Kostić Petrović, Milica Radić

**Affiliations:** 1 Centre for Mental Health Protection, University Clinical Centre Niš, 18000 Niš, Serbia; milannpetkovic@gmail.com (M.N.P.); ivanakostic480@gmail.com (I.K.P.); 2 Faculty of Medicine, University of Niš, 18000 Niš, Serbia; mihajlomarjanovic1010@gmail.com (M.M.); dr.milicaradic@gmail.com (M.R.); 3 Clinics for Oncology, University Clinical Centre Niš, 18000 Niš, Serbia

**Keywords:** chemotherapeutics, working memory, brain fog, structural damage, functional damage

## Abstract

**Highlights:**

**What are the main findings?**

- This review integrates current evidence linking chemotherapy-induced molecular alterations with the neuroanatomical substrates of working memory.
- Different chemotherapeutic agents converge on common mechanisms, including oxidative stress, neuroinflammation, mitochondrial dysfunction, and blood–brain barrier disruption, ultimately affecting neural networks responsible for working memory.

**What are the implications of the main findings?**

- Organizing chemotherapy-associated cognitive impairment according to working memory components provides a novel framework for interpreting clinical neuroimaging and cognitive findings across different malignancies.
- Future multimodal studies combining neuroimaging, molecular biomarkers, and standardized cognitive assessment may facilitate the development of personalized strategies for the prevention and treatment of chemotherapy-associated cognitive impairment.

**Abstract:**

Chemotherapy-associated cognitive impairment (CACI) is a common long-term complication of cancer treatment that predominantly affects attention, executive function, information processing, and working memory. Although considerable progress has been made in understanding its molecular basis, the relationship between chemotherapy-induced neurobiological alterations and the functional organization of working memory remains incompletely understood. This narrative review aims to integrate current evidence on the neuroanatomical organization of working memory, the molecular mechanisms underlying CACI, and neuroimaging findings in patients receiving chemotherapy. The review summarizes current knowledge regarding mitochondrial dysfunction, oxidative stress, neuroinflammation, blood–brain barrier disruption, purinergic signaling, and chemotherapy-specific mechanisms of central nervous system injury. Furthermore, the principal mechanisms by which commonly used chemotherapeutic agents reach or indirectly affect the brain are discussed. Clinical neuroimaging findings from selected malignancies are interpreted within the framework of the four-component model of working memory, including the visuospatial sketchpad, phonological loop, central executive, and episodic buffer. Current evidence indicates that chemotherapy induces structural and functional alterations involving the prefrontal cortex, parietal cortex, hippocampus, insula, anterior cingulate cortex, angular gyrus, and associated neural networks. These changes are accompanied by disturbances in executive control, visuospatial processing, verbal working memory, and multimodal information integration. However, considerable heterogeneity among available studies and the difficulty in distinguishing cancer-related from chemotherapy-induced cognitive impairment remain major limitations. By integrating molecular, neuroanatomical, and neuroimaging evidence, this review proposes a conceptual framework linking chemotherapy-induced brain alterations with dysfunction of specific working memory components. Future longitudinal studies combining multimodal neuroimaging, molecular biomarkers, and standardized neuropsychological assessment are needed to identify chemotherapy-specific patterns of cognitive dysfunction and support the development of personalized preventive and therapeutic strategies for cancer survivors.

## 1. Introduction

Chemotherapy has significantly improved patient survival while reducing quality of life. The concept of a decrease in quality of life being related to changes in the psyche (poor concentration and memory problems) of patients after chemotherapy emerged back in the 1980s and 1990s. One of the pioneering works in this field stressed the need for a better understanding of the problem and a more rigorous scientific approach to its study [1]. In the decades that followed, the term chemotherapy-associated cognitive decline (CACD), or the newer term, chemotherapy-associated cognitive impairment (CACI), was introduced, and there were indications that this side effect of chemotherapy should be considered in the therapy decision-making process [2]. Up to now, collected data on the topic of chemotherapy-associated cognitive decline, though it might appear substantial, is very limited, inconsistent, and inconclusive. Early meta-analyses report small sample sizes, insufficiently sensitive batteries to detect subtle changes in cognitive function induced by chemotherapy, and uncertainty about the onset and extent of cognitive changes [3]. More recent meta-analyses further emphasize the need to address the aforementioned shortcomings and to advance understanding of cognitive disturbances, suggesting that attention, selective attention, verbal memory, and immediate and delayed recall, in both free recall and recognition tasks, are altered in patients receiving chemotherapy [4]. Finally, present-day analyses still require additional findings to substantiate the observed changes in brain connectivity and neural network dynamics following the application of chemotherapeutics [5]. To better understand and study the problem of CACI, the present review aims to reflect on the anatomical and physiological processes underlying the complex functioning of cognition, with a particular focus on working memory, and to examine how these processes are affected by chemotherapeutics. The ultimate goal of this review is to compile and assess the limited contemporary scientific data available on this specific topic, and to potentially connect findings from clinical studies and reports with the known anatomical structures and functional processes associated with working memory.

## 2. Chemotherapeutics

Chemotherapeutics were introduced into medicine following World War II to improve cancer treatment, which had previously relied heavily on surgery and radiotherapy. Later in the 20th century, numerous chemotherapeutics were developed, and nowadays several classes of drugs are available, each targeting different molecular pathways, with treatment protocols updated periodically [6]. It can be said that roughly more than 200 chemotherapeutics are available [7]. Although chemotherapy is widely used and is considered a foundation of cancer treatment, the application is still challenging due to unpredictable response rates and high toxicity (sometimes life-threatening) [8]. Side effects arising from chemotherapeutic agents can be observed as short- or long-term, or as general or organ-specific [7]. Target organs for chemotherapeutic toxicity include the heart, lungs, gastrointestinal tract, and brain.

Cancer treatments often lead to various cognitive impairments, including difficulties with learning, memory, attention, and processing speed. Some pathogenic mechanisms associate these changes with damage to neural progenitor cells in the brain. In particular, both radiation and chemotherapy disrupt hippocampal neurogenesis and compromise the integrity of subcortical white matter, which are considered key mechanisms underlying neurocognitive deficits in cancer survivors [9]. Interestingly, it appears that CACI shares pathways with other brain dysfunctions, as chemotherapy induces oxidative stress in plasma, triggering neuroinflammation and leading to glial and neuronal damage, mitochondrial dysfunction, and eventual cell death [10]. In addition, chemotherapy has been implicated in cerebrovascular dysfunction, which may further contribute to CACD progression [11]. Although there are data indicating changes in brain tissue following exposure to chemotherapeutic agents, there remains a lack of a complete understanding of how these changes reflect the human psyche. The changes could correlate with the type of chemotherapeutic agent used, the route of administration, or the dose and duration of therapy.

## 3. Cognitive Functioning

Cognitive abilities encompass all those that depend on mental functions and enable proper information processing [12]. Mental functions encompass a wide range of abilities, such as perception, attention, memory, intelligence, decision-making, and language skills. These processes cooperate to acquire knowledge, skills, and process information [13]. Cognitive processes begin to develop from the earliest stages of life, with the most intense changes occurring during the first three years, a period marked by the formation of new synapses and the strengthening of existing ones. The school-age period is a crucial stage in the maturation of cognitive functions, particularly in the development of inductive and deductive reasoning [14,15]. An important observation was reported by Katzman and co-workers (1989), where several individuals with advanced Alzheimer’s disease did not exhibit expected cognitive deficits [16]. Building on this observation, the concept of cognitive reserve was introduced and is defined as the brain’s ability to maintain cognitive functioning despite neurological damage. This ability relies on more efficient use of existing neural networks, as well as recruitment of alternative pathways for information processing [17]. There is evidence suggesting that cognitive reserve may potentially play a protective role in preventing cognitive impairment after brain cancer chemotherapy, while patients with lower cognitive reserve had a higher risk of developing Alzheimer’s disease, particularly in the context of specific structural brain characteristics [18,19].

While numerous studies demonstrate the high stability of cognitive abilities, despite this, these functions can be impaired under certain circumstances, including treatments such as chemotherapy [20,21]. Cognitive impairment is one of the most severe long-term consequences of chemotherapy, termed CACI, and significantly affects patients’ daily functioning, social engagement, and quality of life [22]. The prevalence of cognitive dysfunction varies, with 75% of patients reporting cognitive dysfunction during chemotherapy, and about 35% of patients continuing to experience it after the completion of therapy [23]. Most patients report symptoms during the first month after chemotherapy [24]. At the same time, working memory represents one of the most consistently and significantly affected cognitive domains in patients undergoing chemotherapy [25].

## 4. Working Memory

Working memory was considered part of short-term memory; however, it is a complex system that regulates the activity of long-term memory, with attention playing a key role [26]. Multiple brain regions, through mutual interaction, form a unique and integrated system that supports working memory, enabling information to be temporarily retained and actively manipulated, thereby facilitating the performance of more complex cognitive actions such as reasoning, comprehension, and learning [27]. The functioning of working memory involves the coordinated activity of multiple brain regions, with activation patterns varying according to the modality of information being processed [28]. Verbal and auditory information activate Broca’s and Wernicke’s areas in the left hemisphere [29], whereas visuospatial information is processed in the parietal lobe of the right hemisphere, which serves as a workspace for sensory and perceptual integration [30]. The intraparietal sulcus (IPS) is a crucial component of visuospatial working memory, serving as the primary storage site for this information [31]. In this context, the prevailing opinion is that the prefrontal cortex (PFC), specifically the dorsolateral prefrontal cortex (DLPFC), has a dominant role through numerous connections with occipital, temporal, and parietal regions, as well as subcortical structures [32]. The DLPFC contributes significantly to higher cognitive functions, maintaining and manipulating working memory content, and exerting “top-down” control over the IPS to increase working memory capacity [33,34]. Under normal conditions, the IPS’s capacity is regulated by intrinsic excitatory and inhibitory neural circuits [35], but as cognitive demands increase, activation of the DLPFC provides extrinsic excitatory input that overcomes internal inhibition [34].

The activity of these different brain structures changes across the phases of working memory, with the basal ganglia, thalamus, and caudate nucleus being particularly involved in the encoding phase, while the DLPFC and parietal lobes dominate during the retrieval phase [36]. Notably, the basal ganglia help direct attention to relevant information by inhibiting distractors [37], while the anterior cingulate cortex serves as a regulator of attention and evaluates the need for behavioral change [38]. The stability of working memory relies on the PFC, whose activity is directly linked to cortical dopamine originating from the mesocortical pathway and basal ganglia [39]. Since working memory has limited capacity and high energy demands, dopamine is thought to facilitate its use, making it less demanding and increasing willingness to exert effort on difficult tasks [40,41,42,43,44,45]. According to the multicomponent model proposed by Baddeley, working memory consists of four interacting components—the central executive, phonological loop, visuospatial sketchpad, and episodic buffer—which together support the temporary storage and manipulation of information [46].

### 4.1. Visuospatial Sketchpad

The visuospatial sketchpad is a part of working memory whose primary role is the processing and maintenance of visual and spatial information, which is important for visual imagination, spatial orientation, and understanding visual representations [47]. The visual component is responsible for processing image and shape structures and is connected to the occipital cortex, primarily with the V1 region [48]. The spatial component plays an important role in processing information about position, movement, and orientation in space and is linked to the functioning of the parietal lobe of the predominant right hemisphere [49,50]. The capacity of the visuospatial sketchpad is limited, and information is held for only a short time, making it a key limiting factor in tasks that require temporary retention [51].

### 4.2. Phonological Loop

The phonological loop is responsible for the temporary maintenance and processing of verbal and auditory information through two interacting components: the phonological store and the subvocal rehearsal process [52,53]. Its limited capacity contributes to short-term verbal information retention and manipulation [54]. Using fMRI, it has been observed that the subvocal rehearsal system and phonological store activate different regions of the cerebral cortex. Activation of the auditory rehearsal system engaged the primary posterior part of the inferior frontal gyrus (pIFG), the supplementary motor area (SMA), the cerebellum, as well as the ventral premotor cortex (PM). In contrast, the phonological store predominantly activates parts of the parietal cortex, such as the supramarginal gyrus (SMG), the intraparietal sulcus (IPS), and the parietal operculum, as well as the area Spt (Sylvian parietal-temporal area). Transcranial magnetic stimulation suggests that the pIFG has a crucial role in the subvocal system, which is activated even with non-verbal stimuli, while the left SMG is responsible for transient storage of both verbal and non-verbal stimuli [55].

### 4.3. Central Executive

The central executive is a crucial part of working memory that manages the visuospatial sketchpad and phonological loop [26], but it is also closely linked to attention control and retrieving information from long-term memory into working memory [56]. Neuroimaging studies have demonstrated that it primarily depends on the DLPFC and its interactions with the posterior parietal cortex, i.e., it depends on the frontoparietal control network [57]. Additional contributing structures include the anterior cingulate cortex (involved in conflict monitoring and error detection) and basal ganglia circuits (which regulate the updating and gating of information). At the neurochemical level, efficient central executive performance is strongly modulated by dopaminergic and noradrenergic neurotransmission within PFC networks [58].

### 4.4. Episodic Buffer

The episodic buffer is a relatively new component of working memory whose function remains incompletely understood [59]. It is considered an independent storage system, and its role is to integrate information from different modalities into single and coherent mental representations [60]. The process of encoding multiple features of an object activates the episodic buffer, and studies indicate that introducing a second task during encoding disrupts the linking process, implying that the episodic buffer is an independent storage system whose function depends on object-oriented attention [61].

The parietal cortex has long been considered to play a primary role in visuospatial memory and sensorimotor functions [62]; on the other hand, numerous studies have observed significant activity in this region during episodic memory retrieval [63]. Particular attention has been focused on the angular gyrus (AG), for which there is evidence that it participates in the representation and maintenance of retrieved episodic information [64]. Its activation increases in proportion to the number of retrieved details, suggesting a possible role in integrating different elements of memory into a coherent whole. Based on all these findings, it is hypothesized that the AG may represent the neuroanatomical substrate of the episodic buffer, that is, the system responsible for the temporary maintenance and integration of multimodal information during working memory [65].

## 5. Possible Pathophysiological Mechanisms of Chemotherapy-Associated Cognitive Impairment (CACI)

Chemotherapy-associated cognitive impairment (CACI), also known as “brain fog” or “chemo-brain”, is an insufficiently understood condition characterized by cognitive deficits that develop during or after chemotherapy and which may persist for several years following treatment [66,67]. From this perspective, memory, concentration, information processing speed, and executive functions are primarily affected in patients with CACI [68]. The most common and frequent cognitive difficulties experienced by patients after chemotherapy include a sense of mental “fog”, particularly when recalling words and names. Also, there is impaired attention and concentration ability, reduced ability to encode and retain new information, challenges in performing everyday activities, and diminished capacity for multitasking or handling simultaneous tasks [69]. Cognitive impairment may often remain unrecognized, yet it can substantially reduce the quality of life of cancer survivors [70].

Potential risk factors for the development of CACI in patients undergoing chemotherapy are considered to include genetic variants of genes encoding proteins and enzymes that regulate dopaminergic transmission in the frontal cortex or the central metabolism of chemotherapeutic agents via catechol-o-methyl transferase or apolipoprotein E4 (APOE4) [71]. It has been demonstrated that APOE4 intensifies the microglial inflammatory response, thereby directly contributing to the progression of hippocampal damage (promoting hippocampal neurodegeneration) and the impairment of cognitive function [72]. Some studies indicate that stress reduction, anxiety alleviation, and mood improvement can lead to significant improvements in both subjective and objective cognitive functioning [73]. “Chemo-brain” also shares molecular mechanisms with other degenerative brain disorders, including disrupted NAD^+^ metabolism, mitochondrial dysfunction, and persistent inflammatory responses. Neuroinflammation and reactive oxygen species (ROS), which are generated after the administration of chemotherapeutic agents, further accelerate the progression of cognitive impairment [74,75].

Mitochondria play a fundamental role in maintaining nerve cell integrity and function, given their essential roles in ATP production and calcium homeostasis [76]. Under aerobic conditions, mitochondria synthesize ATP through oxidative phosphorylation via the electron transport chain, with ROS generated at complexes I and III, which are the major sources of ROS [77]. Dysfunction of the mitochondria and the electron transport chain results in the accumulation of ROS, which damages mitochondrial DNA, proteins, and lipids, leading to mitochondrial content leakage and their eventual fragmentation [78]. Oxidative stress induced by chemotherapeutics disrupts intracellular calcium homeostasis in microglia, weakening the mitochondrial capacity to buffer calcium [79]. This imbalance further exacerbates excitotoxicity by dysregulating calcium-dependent enzymes, such as CaMKII and CREB, thereby directly impairing synaptic plasticity and long-term potentiation [80]. Since intense synaptic activity imposes high energy demands that rely on local ATP synthesis, its deficit directly impairs critical processes such as action potential maintenance and synaptic vesicle recycling, which, under conditions of impaired mitochondrial homeostasis, leads to the energetic collapse of presynaptic terminals and the loss of synaptic transmission [81].

Although most chemotherapeutic agents do not cross the blood–brain barrier (BBB), a potential model for the development of neuroinflammation, the central mechanism underlying chemo-fog, suggests that elevated peripheral drug levels underlie this condition [82]. Elevated levels of pro-inflammatory cytokines in the blood, which can cross the BBB, disrupt neural homeostasis by stimulating microglial activation and the production and release of pro-inflammatory cytokines, such as tumor necrosis factor α (TNF α) and interleukin-6 (IL-6), and suppressing anti-inflammatory cytokines, such as IL-4 and IL-10, which leads to an alteration in neuronal plasticity [83,84]. Cytokine-induced alterations in neuronal plasticity include abnormal dendritic spine morphology and branching, as well as other structural changes and reduced neuronal density [82]. Elevated levels of central cytokines have been associated with widespread reductions in gray and white matter density, disruption of neural networks, and changes in hippocampal volume and prefrontal and temporal cortex metabolism (6 months after chemotherapy), all of which are fundamental to important cognitive functions [85]. Moreover, Toll-like receptor 4 (TLR4) may have a crucial role in disrupting BBB integrity, as it is activated by chemotherapy-induced oxidative stress leading to TNFα synthesis [86,87]. Furthermore, TLR4 activation stimulates the NF-κB signaling pathway, a critical regulator of inducible nitric oxide synthases and proapoptotic proteins (BAX, p53), leading to mitochondrial dysfunction and the release of cytochrome c [88]. Collectively, these processes induce neuronal apoptosis.

CACI has also been associated with a dysregulation in purinergic signaling, whereby increased adenosine release in the hippocampus activates A2A receptors, leading to neuroinflammation and impaired synaptic plasticity [89,90]. Such excessive activation of adenosine receptors disrupts LTP mechanisms, thereby directly contributing to learning and memory deficits in patients undergoing cytostatic therapy [91]. Although studies on the impact of chemotherapy on purinergic receptor activation in patients with cancer are limited, experimental studies indicate that A2A purinergic receptors influence neuronal processes under both physiological and pathological conditions [92]. These receptors regulate neuroplasticity and synaptic transmission under physiological conditions, and their excessive activation in pathological states can contribute to neuroinflammation, excitotoxicity, and the onset of cognitive impairment [93]. In a mouse model of cisplatin-induced cognitive deficit, increased expression of A2A receptors and their downstream signaling molecules was observed in the hippocampus, while A2A receptor blockade led to improved memory function and improved dendritic morphology of newly formed neurons [89].

Using modern neuroimaging methods, we have gained insights into the structural and functional changes in individuals with CACI [94]. Using magnetic resonance imaging (MRI), reduced gray matter volume in the frontal, temporal, and parietal lobes, as well as reduced cortical thickness in the prefrontal, parietal, and insular cortices, were observed [95]. A few years later, using diffusion magnetic resonance imaging and voxel-based analysis, reduced fiber density (FD) in the splenium of the corpus callosum, which connects the temporal and occipital regions of both hemispheres and plays a key role in language, visuospatial information transfer, and behavioral regulation, was identified [96].

Finally, it should be noted that in patients with colorectal carcinoma, there may be a certain degree of cognitive impairment which occurs prior to chemotherapy, and that this process is further accelerated by the administration of chemotherapy [97]. A similar issue has been noted in patients with breast cancer and non-small-cell lung cancer, where a pre-existing statistically significant decrease in working memory at baseline was observed [98]. This raises the question whether cognitive decline can be associated with molecules produced by the cancer itself, and if the administration of chemotherapeutics intensifies cognitive deterioration.

**Figure 1:**
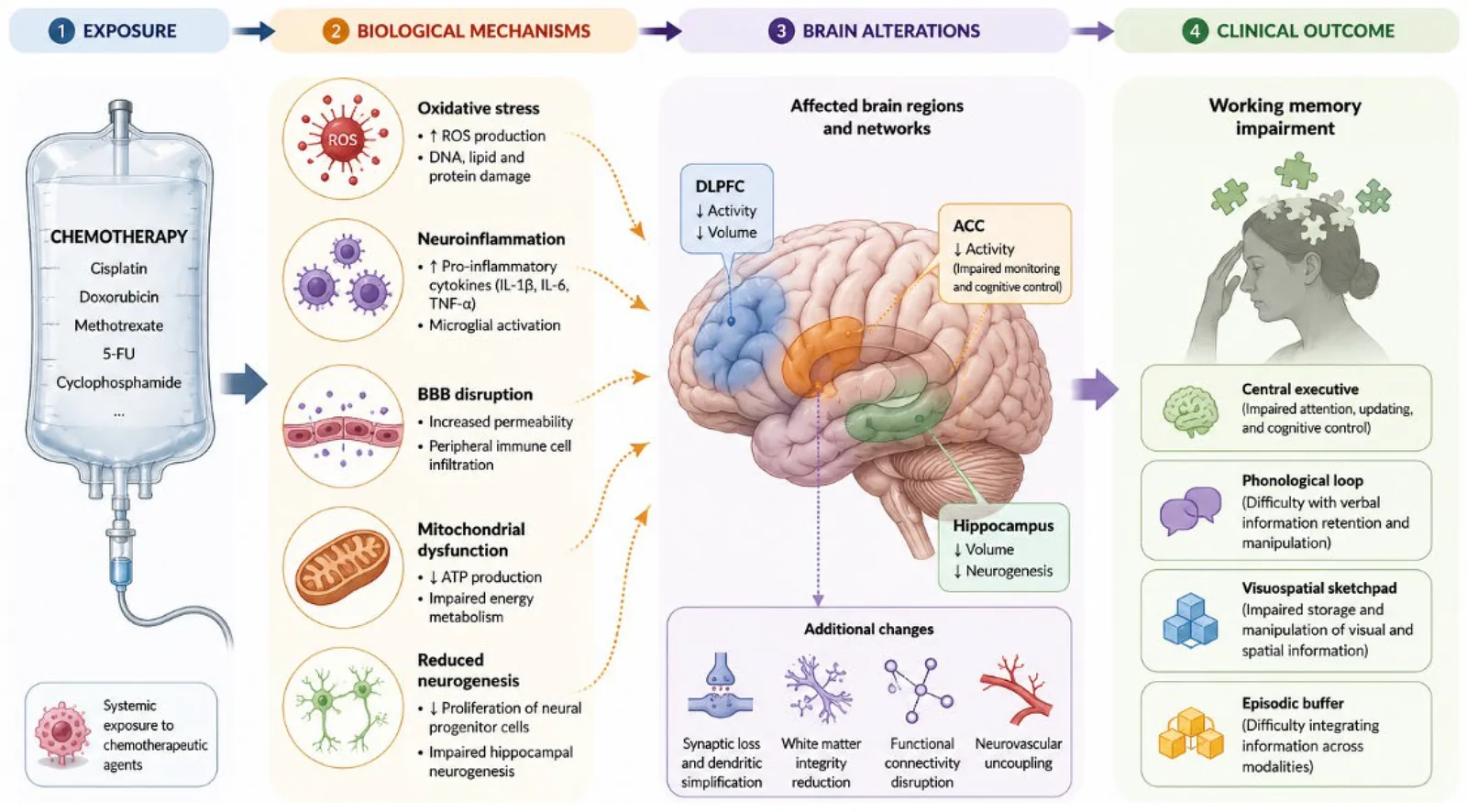
Proposed mechanisms underlying chemotherapy-induced working memory impairment. Chemotherapeutic agents, including platinum compounds, anthracyclines, antimetabolites, and alkylating agents, initiate multiple interconnected biological processes, including oxidative stress, neuroinflammation, blood–brain barrier (BBB) disruption, mitochondrial dysfunction, and reduced neurogenesis. These mechanisms contribute to neuronal and glial dysfunction, leading to structural and functional alterations in brain regions involved in working memory, particularly the dorsolateral prefrontal cortex (DLPFC), anterior cingulate cortex (ACC), hippocampus, and white matter networks. The resulting disruption in fronto-hippocampal connectivity and neural network integrity ultimately impairs the major components of working memory, including executive control, phonological processing, visuospatial processing, and information integration. Abbreviations: BBB, blood–brain barrier; DLPFC, dorsolateral prefrontal cortex; ACC, anterior cingulate cortex; ROS, reactive oxygen species.

The figure summarizes the principal mechanisms discussed throughout this review, and illustrates the proposed cascade linking chemotherapy exposure to working memory dysfunction (Figure 1).

In addition to direct neurotoxic effects, systemic immune activation and peripheral inflammatory responses secondary to chemotherapy-induced cell death may contribute to cognitive dysfunction, including working memory impairment. Comparative studies evaluating conventional cytotoxic chemotherapy and non-cytotoxic anti-cancer therapies may further clarify the relative contribution of systemic immune signaling to CACI.

Susceptibility to CACI is likely influenced by an interaction between treatment-related and patient-related factors. Treatment-related factors may include the specific chemotherapeutic agent, route of administration, cumulative exposure, dose, and duration of treatment. Patient-related susceptibility may involve genetic variants affecting dopaminergic transmission or chemotherapeutic metabolism, particularly COMT, as well as the APOE4 genotype [71]. APOE4 may enhance microglial inflammatory responses and thereby contribute to hippocampal neurodegeneration and cognitive dysfunction [72]. In contrast, higher baseline cognitive reserve and education level may exert a partially protective effect by enabling more efficient use of existing neural networks or recruitment of alternative pathways, whereas lower cognitive reserve has been associated with greater cognitive decline following chemotherapy [18]. Potentially modifiable psychological factors may also influence cognitive outcomes, as reductions in stress and anxiety and improvements in mood have been associated with better subjective and objective cognitive functioning [73]. Nevertheless, evidence for interventions administered before, during, or after chemotherapy that specifically prevent or restore working memory dysfunction remains limited, and no established intervention can currently be recommended on the basis of the studies included in this review.

## 6. How Do Chemotherapeutics Reach the Brain?

Although most chemotherapeutics do not cross the BBB, certain agents are intentionally designed to penetrate it and target primary or metastatic brain tumors [99]. In a recent study, a summary was presented showing that the bioavailability and cerebrospinal concentrations of chemotherapeutics vary with molecular characteristics [100]. Although some agents cross the BBB more rapidly than others, their accumulation is less frequent than one might expect owing to rapid drug clearance mechanisms [100]. Here, we only explain some mechanisms for the most prominent and frequently used drugs, with the most clearly elucidated ways of transport across the BBB (Table 1).

The BBB is the principal anatomical and functional obstacle limiting the entry of chemotherapeutic agents into the central nervous system. It consists of specialized endothelial cells connected by tight junctions and supported by pericytes and astrocytes, while ATP-dependent efflux transporters, including P-glycoprotein (ABCB1), breast cancer resistance protein (BCRP), and multidrug resistance-associated proteins (MRPs), actively remove many chemotherapeutic agents from the brain. Consequently, only compounds with favorable physicochemical properties or those utilizing endogenous transport systems can achieve measurable brain concentrations [101].

Cyclophosphamide and its active metabolites have limited penetration across the intact BBB and, being small, water-soluble molecules, primarily rely on passive diffusion. Thus, if penetration into brain tissue is needed, it has historically been applied in a hyperosmotic solution (mannitol). Due to limited neurotoxicity, it is considered a favorable chemotherapeutic [102]. 5-fluorouracil crosses the BBB more readily than many other chemotherapeutic agents and has been shown in animal studies to impair hippocampal neurogenesis, reduce oligodendrocyte proliferation, and disrupt myelin integrity [103]. Unlike many chemotherapeutic agents, 5-fluorouracil can cross the blood–brain barrier via passive diffusion due to its relatively low molecular weight, thereby reaching the central nervous system and contributing to neurotoxicity [103]. Platinum-based chemotherapeutic agents, including cisplatin, carboplatin, and oxaliplatin, are highly polar compounds that are generally unable to cross the BBB. However, evidence suggests that these drugs can penetrate the BBB via copper transporter 1 (CTR1) [104]. CTR1 is a high-affinity transmembrane copper transporter that forms a pore within the cell membrane, whose inner surface contains methionine-rich and cysteine-rich motifs capable of binding copper ions, as well as platinum-containing compounds such as cisplatin [105]. Cisplatin is transported via the transporter through sequential transchelation reactions, during which platinum is sequentially transferred between stacked methionine rings [104]. Carboplatin shares similar physicochemical characteristics with cisplatin and demonstrates limited BBB penetration. Transport through CTR1 has also been proposed, although its lower chemical reactivity results in reduced neurotoxicity compared with cisplatin [104]. Oxaliplatin has minimal penetration across the intact BBB, but experimental evidence suggests that CTR1 contributes to its cellular uptake. Neurotoxicity is thought to result predominantly from mitochondrial dysfunction, oxidative stress, and neuroinflammatory mechanisms rather than from extensive CNS accumulation [106]. Finally, there are some drugs, such as paclitaxel, that are incapable of penetrating the intact BBB and enter brain tissue, yet they are associated with cognitive changes in patients receiving treatment [107]. The ability of this drug to enter brain tissue is mediated by indirect damage to the BBB via cascades involving oxidative stress and inflammatory cytokines, which, together with toxic drug metabolites, damage nerve cells, thereby provoking cognitive decline [108]. Despite negligible BBB penetration, in animal studies, doxorubicin induces peripheral oxidative stress and cytokine release, particularly TNF-α, which subsequently activates microglia and promotes central neuroinflammation [109]. This indirect mechanism is considered one of the major pathways responsible for doxorubicin-induced cognitive impairment.

**Table 1:** Blood–brain barrier penetration and mechanisms of neural toxicity of selected chemotherapeutic agents.

Drug [Reference]	BBB Penetration	Main Transport Mechanism	Main Mechanism of Cognitive Injury
Cyclophosphamide [102]	Limited	Passive diffusion	Oxidative stress
Cisplatin [104]	Very limited	CTR1	ROS, mitochondrial dysfunction
Carboplatin [104]	Very limited	CTR1	Neuroinflammation
Oxaliplatin [106]	Very limited	CTR1	Peripheral neuropathy, ROS
5-FU [103]	Moderate	Passive diffusion	Impaired neurogenesis
Doxorubicin [109]	Minimal	Indirect (cytokines)	TNFα-mediated inflammation
Paclitaxel [108]	Very limited	Indirect BBB dysfunction	Microglial activation

## 7. Insights from Experimental Animal Models

Non-human studies provide important evidence linking chemotherapy exposure to both neurobiological alterations and behavioral consequences. Unlike clinical studies, experimental animal models allow greater control over the chemotherapeutic agent, dose, treatment schedule, and timing of behavioral assessment, while also enabling direct examination of brain tissue. Rodent research indicates that chemotherapy may disrupt hippocampal neurogenesis, dendritic morphology, synaptic plasticity, mitochondrial function, white matter integrity, and neuroinflammatory signaling, thereby providing plausible biological explanations for changes in working memory, learning, attention, and other cognitive behaviors [9,10,11]. For example, in a mouse model of cisplatin-induced cognitive impairment, increased hippocampal A2A receptor signaling was accompanied by memory dysfunction and altered dendritic morphology, whereas pharmacological blockade of these receptors improved memory performance and neuronal morphology [89]. Experimental findings also indicate that 5-fluorouracil can impair hippocampal neurogenesis, reduce oligodendrocyte proliferation, and disrupt myelin integrity [103], while paclitaxel and doxorubicin may induce cognitive alterations through oxidative stress, peripheral cytokine release, blood–brain barrier dysfunction, and microglial activation [107,108,109]. Thus, animal studies support a causal relationship between chemotherapy exposure, neural injury, and behavioral impairment and help identify potentially modifiable molecular pathways. Nevertheless, their translation to patients requires caution because experimental doses, treatment schedules, behavioral paradigms, and the frequent use of animals without tumors do not fully reproduce the complexity of clinical cancer treatment.

## 8. Changes in Working Memory in Patients with Cancers of Different Localization

Changes in cognitive functioning have been examined in patients with cancers of different localization, since chemotherapeutic and radiotherapeutic approaches differ between cancers. With this in mind, cancers localized in the CNS produce cognitive impairment and it is almost impossible to distinguish the precise reason for it. Consequently, cancers with distant metastases in the CNS are not discussed in this section for the same reason. Only selected malignancies were included in this review section, as they represent the cancer types for which the strongest and most consistent evidence regarding chemotherapy-associated working memory impairment is currently available. This approach was adopted to provide a focused synthesis of the most recent literature rather than an exhaustive overview of all malignancies. Although this review discusses evidence across different cancer types, the available literature and the studies included predominantly involve adult cancer patients. Therefore, the discussion primarily reflects findings from adult populations unless otherwise stated.

The occurrence of CACI has been most thoroughly investigated in breast cancer patients [110], which has been, from the construct of this concept in the 1990s, highly investigated and followed [20]. From 29 neuroimaging studies, it has been concluded that in patients with breast cancer after chemotherapy, there is a significant reduction in gray matter in regions such as the orbitofrontal cortex, anterior cingulate cortex, insula, cerebellum, and rolandic operculum [110]. Also, there is significant functional hypoactivation in the rolandic operculum and insula [110], the dysfunction of which can be traced to the impairment of attention control, cognitive flexibility, conflict monitoring, and executive functioning, given the established role of the insula in reorienting attention, switching between cognitive states [94,111]. Interestingly, although CACI is suggested to be associated with different brain regions, the changes in these regions (e.g., temporal cortex and hippocampus) or in the connectivity of structures (parietal and prefrontal areas) were not found to be significant in the meta-analysis, suggesting that this might arise from the larger scale of the study [110]. On the other hand, some additional structures, such as the cerebellar crus (part of cognitive cerebellum) and rolandic operculum (involved speech production), that are indirectly associated with cognitive functioning are altered in patients with BC [110]. Generally, some early longitudinal studies showed that memory deficit and executive functions are impaired mainly in patients with breast cancer after chemotherapy [112]. As mentioned, cognitive reserve might be connected with lesser CACI, and a study conducted in breast cancer patients reported that patients with lower baseline cognitive reserve exhibited greater declines in processing speed after chemotherapy [113].

One of the first prospective longitudinal studies conducted in 2019 examined neurocognitive network functioning and correlated the changes with the psychological findings in patients with gynecological malignancies [114]. The results indicated that the functional hubs associated with cognitive dysfunction, or, to be more precise, network resilience and regulation of information flow, located in the left and right insula, middle temporal gyrus, superior temporal gyrus, left hippocampus, and parahippocampal gyrus, were altered compared to the control [114]. The mentioned structures are part of all four processes associated with working memory. Due to the fact that chemotherapeutics alter cell metabolism to different extents, a study was conducted in patients with cervical cancer, within half a year post-therapy, in order to establish the impact of therapy on 18F-fluorodeoxyglucose metabolism [115]. Interestingly no changes in brain metabolic areas were found in patients who had undergone chemotherapy only, but in cases when chemotherapy was combined with radiotherapy, there was a decrease in glucose metabolism in frontotemporal regions, which can be associated with cognitive difficulties [115].

As aforementioned, gastrointestinal cancers have been associated with some cognitive changes [97], with them being more intensified after chemotherapy [97,116]. In surviving colorectal cancer patients (a year from diagnosis), a hyperactivity in the temporal regions and disrupted default mode network has been connected with cognitive decline [117]. One of the most recent discoveries in patients with gastrointestinal malignancies identified significant alterations in the frontoparietal and sensorimotor networks compared to healthy individuals, which are thought to be connected with cancer-related fatigue [118]. Finally, dysregulation in gut microbiota and dietary intake seen in cancer patients has been suggested to impact cognitive functioning [119].

Similar to patients with gastrointestinal malignancies, changes in cognition have been seen in patients with lung cancers prior to chemotherapy [120]. Altered visuospatial/executive function and delayed memory have been connected with disease advancement [120]. Also, in lung cancer patients receiving either cisplatin- or carboplatin-based chemotherapy over 3 to 6 months, a significant regional homogeneity (ReHo) in the left precuneus, right central operculum, right inferior parietal lobule, and right superior occipital gyrus, but lower in the left inferior frontal gyrus and right MFG, have been found, suggesting clear existence of neurological foundations for CRCI [121].

Children and adolescents represent a unique population in the context of chemotherapy-associated cognitive impairment because the developing brain is particularly susceptible to treatment-related neurotoxicity. Although this review primarily focuses on adult cancer patients, evidence from pediatric oncology demonstrates that childhood cancer survivors frequently experience persistent cognitive deficits that may continue for years after treatment completion. These impairments are particularly well-documented in survivors of acute lymphoblastic leukemia (ALL), even in the absence of cranial irradiation, suggesting that chemotherapy itself contributes to long-term neurocognitive sequelae. Compared with adults, pediatric patients face additional challenges because chemotherapy may interfere with ongoing brain maturation rather than affect fully developed neural networks. Consequently, cognitive deficits may become more apparent over time as educational and executive demands increase during adolescence and early adulthood. Long-term neuropsychological follow-up and early cognitive rehabilitation are therefore important components of survivorship care, with the aim of minimizing the impact of treatment-related cognitive impairment on educational attainment, social functioning, and quality of life.

Lymphomas frequently occur in young age, and thus aggressive treatment protocols might affect the brain during a sensitive period of development, potentially preventing/altering development [122]. In patients with non-Hodgkin’s lymphoma, there has also been observed a decrease in cognitive capacity estimated through various cognitive tasks, before chemotherapy and within 2 weeks of two chemotherapy courses, as well as a significant decrease in visuospatial ability, but without any morphofunctional analysis to confirm such findings [123]. A recent systematic review showed a limited number of adequately designed publications examining the alterations in cognitive function of patients with different lymphomas [122]. Some of the conclusions from such studies are that there are discrepancies in detecting and reporting cognitive decline in patients that received therapy for classical Hodgkin’s lymphoma. Namely, the patients with clinically observable cognitive decline did not report more difficulties than other patients [122]. In the most recent publication, aiming to overcome the suggested downfalls of the studies in patients with CACI, a study design examining changes in patients’ brain functioning prior and after chemotherapy treatment was used [124]. Patients (*n* = 9) diagnosed with aggressive lymphoma underwent neuropsychological assessment and anatomical MRI before and 6–8 weeks after chemotherapy [124]. In a limited sample, they reported changes in executive function and verbal memory (*n* = 3) as well as changes to the infranormal cortical thickness and/or surface area in the superior frontal gyrus (*n* = 7), and only in a single case in ACC [124].

## 9. Alterations in Specific Brain Regions Associated with Working Memory

Back in the early 2000s, neuroimaging studies using MRI examined the relationship between the application of different chemotherapy regimens (uracil, cyclophosphamide, methotrexate, and 5-fluorouracil) in patients with cancer [125]. The results indicate a reduction in white and gray mass after 1 year, indicating that there is a clear connection between white matter integrity, cognitive impairment, and chemotherapy [125]. In the next section, we selected a few representative publications with results directly or indirectly showing how the brain structure and function of specific regions which are associated with the process of working memory are altered. Furthermore, these findings are brought in connection with specific chemotherapeutic agents (where possible) which may be linked to specific damage to some structures.

### 9.1. Chemotherapeutic-Induced Visuospatial Impairment

Chemotherapy has been shown to particularly affect visuospatial working memory, which depends on the IPS and DLPFC. Following the administration of chemotherapeutic agents such as doxorubicin-cyclophosphamide-paclitaxel, docetaxel-doxorubicin-cyclophosphamide, and doxorubicin-cyclophosphamide, increased neuronal activity detected by functional magnetic resonance imaging (fMRI) has been observed in the PFC, likely reflecting a compensatory mechanism arising from reduced capacity in the parietal cortex [126]. Given that previous studies have primarily examined changes in visuospatial memory in patients with breast cancer, studies analyzing the impact of chemotherapy on this cognitive function in other malignancies were also considered. The long-term (2 to 7 years post-therapy) cognitive effects of BEP chemotherapy (bleomycin, etoposide, and cisplatin) in a cross-sectional study involving patients with testicular cancer have also been assessed [127]. Assessment of visuospatial memory using the Rey Complex Figure Test did not show a statistically significant impairment compared to patients who had not received chemotherapy. Such findings suggest that the effects of chemotherapy on visuospatial memory may vary depending on the therapeutic protocol used, which raises the possibility that different cytostatics produce different neurotoxic effects. Given that in most studies showing visuospatial memory changes in breast cancer patients, and that the therapy was based on doxorubicin, it is possible that different chemotherapy regimens contribute to the observed differences in cognitive outcomes.

Some studies suggest that alterations in visuospatial working memory already exist in patients with breast cancer before chemotherapy, including slower reaction time, lower accuracy, and increased activation of working memory-related brain regions [128]. However, post-chemotherapeutic studies have demonstrated more pronounced cognitive decline accompanied by both functional and structural brain changes, particularly within frontal and hippocampal networks. These findings suggest that baseline cognitive and functional brain alterations may precede treatment, and should therefore be considered when interpreting chemotherapy-associated cognitive impairment. Similar alterations prior to chemotherapy have been noted in patients with lung cancer. In those subjects, a clear visuospatial/executive function and delayed memory dysfunction has been seen [120]. This is further connected with a reduction in cortical thickness and surface area in frontal, parietal, and temporal lobes [120].

### 9.2. Chemotherapeutics-Induced Phonological Loop Impairment

As aforementioned, the parietal and temporal lobes are involved during activation of the phonological loop [55], while their function is controlled by the central executive. In patients with breast cancer, a more significant increase in activation of the right inferior temporal gyrus while performing cognitive tasks has been observed, compared to a control group. These results correspond with an increase in blood flow of a specific region in these patients even prior to chemotherapy [129].

In the brains of patients with non-small-cell lung carcinoma, treated with therapeutic modalities such as pemetrexed plus carboplatin and paclitaxel plus carboplatin, a significant ReHo in the IFG was observed [94]. The changes were detected using resting-state functional magnetic resonance imaging (rs-fMRI), with ReHo being used as an indicator of local neuronal activity synchronization. Similar results were found in a cross-sectional neuroimaging study of patients with acute myeloid leukemia who were following a therapeutic protocol consisting of D-nucleoside arabinoside cytarabine, C-CAG cytarabine, aclamycin, and granulocyte colony-stimulating factor (D-CAG) chemotherapy [115]. Compared to the control group, a significant decrease was detected in the right prefrontal cortex, particularly in the middle and inferior frontal gyrus pars opercularis, in patients receiving chemotherapy [115]. Local changes in homogeneity in the IFG point to the dysfunction of prefrontal networks involved in working memory and verbal information processing, which might potentially represent the neuroanatomical substrate of the phonological loop disorder in these patients.

### 9.3. Chemotherapeutics-Induced Central Executive Impairment

Since the central executive encompasses several brain structures and their connections, as well as some specific neurotransmitter systems, some study findings will be presented. Elevated ReHo was identified in the right orbitofrontal cortex and left DLPFC, along with changes in white matter integrity, in breast cancer patients receiving chemotherapy [25]. The observed hyperactivation in the mentioned regions suggests a compensatory mechanism reflecting decreased neural processing efficiency after chemotherapy application [25]. Connectivity between one of the basal ganglia, putamen, and cortical structures has been found to be affected in breast cancer patients treated with chemotherapy [130], suggesting that through these changes, attention might be negatively affected. In a recent longitudinal study examining the effects of chemotherapy, a pre- and post-MRI study showed that only in around 10% of patients was there a decrease in gray mass thickness in ACC [124], suggesting that changes in this region alone are unlikely to fully explain CACI. Interestingly, an increase in insular activation in breast cancer patients that received chemotherapy is known to be associated with an increased demand of these processes in managing the phonological loop [129].

Rare experimental studies examining changes in neurotransmission in patients with CACI have been conducted. Some neuroimaging studies show reduced dopamine transporter availability in the dorsal striatum of cancer survivors experiencing CACI, suggesting persistent alterations in the nigrostriatal dopaminergic system [131]. These findings support the hypothesis that dopaminergic dysfunction may contribute to the dysfunction of the central executive through diffusion via the basal ganglia. Cisplatin is one of the chemotherapeutics for which most of the clinical experimental animal and human evidence associated with dopaminergic dysfunction has been collected over the years. However, no direct human study showing loss of dopaminergic neurons has been published, and only indirect evidence is present. Namely, clinical reports of chemotherapy-associated parkinsonism and extensive experimental evidence support the hypothesis that cisplatin may adversely affect dopaminergic neurotransmission through mechanisms involving oxidative stress, mitochondrial dysfunction, and neuroinflammation [132].

### 9.4. Chemotherapeutics-Induced Episodic Buffer Impairment

Although the episodic buffer as a concept has not been directly examined in neuroimaging studies of patients after chemotherapy, the findings suggest dysfunction of the neural structures and network that is crucial for its functioning [133]. The episodic buffer enables the integration of information from the phonological loop, the visuospatial sketchpad, and long-term memory into a unified episodic representation [134]. This process depends on the integrity of the hippocampus and the connected regions of the recollection network, including the parahippocampal gyrus, precuneus, AG, and mPFC. Neuroimaging studies in patients treated with chemotherapy show structural and functional changes in these regions, which may point to a weakened capacity for binding and integrating multimodal elements of experience into a coherent episodic whole [135]. This directly prevents the function of the episodic buffer since it can no longer fluidly integrate visual and verbal details under heavy cognitive loads, leading to mental fog [136].

In a cross-sectional resting-state MRI study, functional changes were found in multiple brain regions including the prefrontal cortex, bilateral middle temporal gyrus, right inferior temporal gyrus, right angular gyrus, left insula, and left nucleus caudatus in patients with breast cancer after chemotherapy [137]. These changes were accompanied by deficits in memory, attention, executive functions, information processing speed, and word retrieval. It is particularly interesting that changes in the right angular gyrus were consistently described in almost all analyzed neuroimaging studies, regardless of the type of malignancy or the MRI methodology applied [137,138,139,140,141]. Since this region is involved in episodic recall, verbal working memory, and information integration through its episodic buffer function, its dysfunction may represent one of the mechanisms underlying cognitive impairments in cancer patients. This consistency in findings, observed in patients with breast, gastric, and colorectal cancer, suggests that the right angular gyrus may represent a common substrate for cognitive impairment associated with malignancy and its treatment.

The temporal course of chemotherapy-associated cognitive and working memory impairment is heterogeneous, and the available evidence does not support describing these alterations as uniformly temporary or permanent. Cognitive difficulties are commonly reported during chemotherapy and in the early period following treatment, while many patients subsequently experience partial or substantial recovery. Nevertheless, approximately 35% of patients continue to report cognitive dysfunction after treatment completion, and in some individuals, CACI may persist for several years [23,66,67]. Longitudinal findings demonstrate cognitive and structural brain alterations within weeks after chemotherapy [124], while reduced regional gray- and white-matter volumes have also been detected one year after treatment [125]. In contrast, long-term assessment of patients treated with BEP chemotherapy did not identify significant visuospatial memory impairment compared with patients who had not received chemotherapy [127]. These apparently inconsistent findings suggest that persistence may depend on the treatment regimen, cumulative exposure, cancer population, baseline vulnerability, and the cognitive domain and assessment method used. Furthermore, the presence of working memory differences before chemotherapy in some patients complicates the attribution of persistent deficits solely to treatment [128]. Therefore, current evidence supports a variable trajectory ranging from recovery to prolonged impairment, but remains insufficient to establish that chemotherapy-associated working memory alterations are permanent.

## 10. Study Advantages and Limitations

Despite the fact that the present review is narrative in nature, it provides significant information for and a critical approach to modern understandings of the relatively broad and heterogeneous topic of cognitive decline in cancer patients. For the first time, this study explains the changes in brain regions related to specific anatomical and functional correlates of working memory seen in patients after chemotherapeutic administration, which is a major strength of this narrative review. Furthermore, it integrates evidence from molecular biology, blood–brain barrier physiology, neuroanatomy, neuroimaging, and cognitive neuroscience, thereby linking experimental findings with clinically relevant cognitive outcomes. This multidisciplinary approach offers a comprehensive overview of the mechanisms that may underlie working memory dysfunction in patients undergoing chemotherapy.

On the other hand, one can argue that there is a lack of a systematic and structural search of the libraries which could yield a final result on the topic. Bearing in mind that the study encompasses publications explaining cognitive changes in patients with cancers of different localization, it would be almost impossible to include every publication without omission of some valuable findings that do not fit into a search. This would also lead further from the basic topic of the review which is to emphasize and bring together preliminary data on the changes in brain regions associated with working memory. Another important limitation of the currently available literature is the substantial heterogeneity in chemotherapy protocols, cumulative drug doses, treatment duration, administration schedules, and timing of neuropsychological assessment after chemotherapy, which precludes meaningful comparison between studies and limits the possibility of identifying chemotherapy-specific patterns of cognitive impairment. Moreover, much of the current literature is derived from breast cancer cohorts, whereas data on many other malignancies remain relatively limited. One could also argue that there is lack of a mention of a specific drug which might lead to CACI; however, also bearing in mind that treatment protocols rarely include less than two chemotherapeutics (frequently more than three), it would be almost impossible to trace back the specific effect to one substance. Another limitation is the substantial heterogeneity in patient demographics, particularly age and sex distribution, which precludes meaningful conclusions regarding potential differences in susceptibility to chemotherapy-induced working memory impairment across different populations. The interpretation of chemotherapy-associated cognitive impairment is further complicated by multiple potential confounding factors, including the underlying malignancy, surgery, radiotherapy, corticosteroid use, concomitant medications, fatigue, anemia, and psychological distress. Finally, many proposed molecular mechanisms are supported primarily by experimental animal studies, and their direct translation to human chemotherapy-associated cognitive impairment requires further clinical validation.

## 11. Conclusions and Future Perspectives

Chemotherapy-associated cognitive impairment is a multifactorial complication resulting from the interplay of oxidative stress, mitochondrial dysfunction, neuroinflammation, blood–brain barrier disruption, and impaired neuroplasticity. Although chemotherapeutic agents differ in their ability to reach the central nervous system and in their mechanisms of neurotoxicity, they ultimately affect interconnected brain regions responsible for working memory, resulting in deficits in attention, executive function, and information processing. This review integrates current evidence linking chemotherapy-induced molecular alterations with the neuroanatomical substrates of working memory, providing a framework for understanding the mechanisms underlying CACI. However, the considerable heterogeneity among available studies and the difficulty in distinguishing cancer-related from chemotherapy-induced cognitive impairment remain important limitations. Future studies should combine longitudinal neuroimaging with age- and sex-related differences (including potential developmental and aging-associated windows of vulnerability), molecular biomarkers, and standardized cognitive assessment to identify chemotherapy-specific patterns of brain injury and develop personalized strategies for the prevention and treatment of CACI. This approach may improve both cognitive outcomes and the quality of life of cancer survivors.

## Data Availability

No new data were created or analyzed in this study. Data sharing is not applicable to this article.

## Abbreviations

ACC	Anterior Cingulate Cortex
AG	Angular Gyrus
APOE4	Apolipoprotein E4
ATP	Adenosine Triphosphate
BAX	BCL2-Associated X Protein
BBB	Blood–Brain Barrier
BC	Breast Cancer
BCRP	Breast Cancer Resistance Protein
BDNF	Brain-Derived Neurotrophic Factor
BEP	Bleomycin, Etoposide, and Cisplatin
CACI	Chemotherapy-Associated Cognitive Impairment
CACD	Chemotherapy-Associated Cognitive Decline
CaMKII	Calcium/Calmodulin-Dependent Protein Kinase II
CNS	Central Nervous System
CREB	cAMP Response Element-Binding Protein
CTR1	Copper Transporter 1
D-CAG	Cytarabine, Aclamycin, and Granulocyte Colony-Stimulating Factor Regimen
DLPFC	Dorsolateral Prefrontal Cortex
FD	Fiber Density
fMRI	Functional Magnetic Resonance Imaging
IFG	Inferior Frontal Gyrus
IL	Interleukin
IPS	Intraparietal Sulcus
LTP	Long-Term Potentiation
MFG	Middle Frontal Gyrus
mPFC	Medial Prefrontal Cortex
MRP	Multidrug Resistance-Associated Protein
MRI	Magnetic Resonance Imaging
NAD^+^	Nicotinamide Adenine Dinucleotide
NF-κB	Nuclear Factor Kappa B
NSCLC	Non-Small Cell Lung Cancer
p53	Tumor Protein p53
PFC	Prefrontal Cortex
pIFG	Posterior Inferior Frontal Gyrus
PM	Premotor Cortex
ReHo	Regional Homogeneity
ROS	Reactive Oxygen Species
rs-fMRI	Resting-State Functional Magnetic Resonance Imaging
SMA	Supplementary Motor Area
SMG	Supramarginal Gyrus
Spt	Sylvian Parietal-Temporal Area
TLR4	Toll-Like Receptor 4
TMS	Transcranial Magnetic Stimulation
TNF-α	Tumor Necrosis Factor Alpha
V1	Primary Visual Cortex
5-FU	5-fluorouracil
